# A Novel KCNQ2 Variant in a Patient with a Combined Tremor Syndrome

**DOI:** 10.5334/tohm.887

**Published:** 2024-05-09

**Authors:** Giulia Paparella, Eleonora Galosi, Emanuele Cerulli Irelli, Luca Angelini, Daniele Birreci, Davide Costa, Martina De Riggi, Antonio Cannavacciuolo, Andrea Truini, Matteo Bologna

**Affiliations:** 1 IRCCS Neuromed, Pozzilli (IS), Italy; 2 Department of Human Neurosciences, Sapienza University of Rome, Italy

**Keywords:** tremor, movement disorders, KCNQ2, kinematics, skin biopsy

## Abstract

**Background:**

Tremor disorders have various genetic causes.

**Case report:**

A 60-year-old female with a family history of tremor presented a combined tremor syndrome, transient episodes of loss of contact and speech disturbances, as well as distal painful symptoms. Genetic screening revealed a novel heterozygous missense variant in the KCNQ2 gene.

**Discussion:**

The KCNQ2 protein regulates action potential firing, and mutations in its gene are associated with epilepsy and neuropathic pain. The identified variant, although of uncertain significance, may disrupt KCNQ2 function and also play a role in tremor pathogenesis. This case highlights the importance of genetic screening in combined tremor disorders.

Tremor, an involuntary, rhythmic, oscillatory movement of a body part, is one of the prevalent movement disorders [1]. Various genetic defects have been implicated in tremor etiology [2,3,4,5], with familial patterns often suggesting a complex heritability [2]. We present a case of combined tremor syndrome, initially diagnosed as essential tremor (ET), in a patient with burning feet and epileptic-like manifestations carrying a novel missense variant in the KCNQ2 gene.

## Case Report

A 60-year-old female patient was referred to the Movement Disorders outpatient clinic, having previously received an ET diagnosis several years earlier. She reported upper limb tremors since childhood, with additional voice and head tremors onset in young adulthood. The patient’s father had experienced head tremor from an early age and was diagnosed with Parkinson’s disease after 60; her paternal aunt had upper limbs dystonic tremor. The patient provided written informed consent to the study.

**Figure 1:**
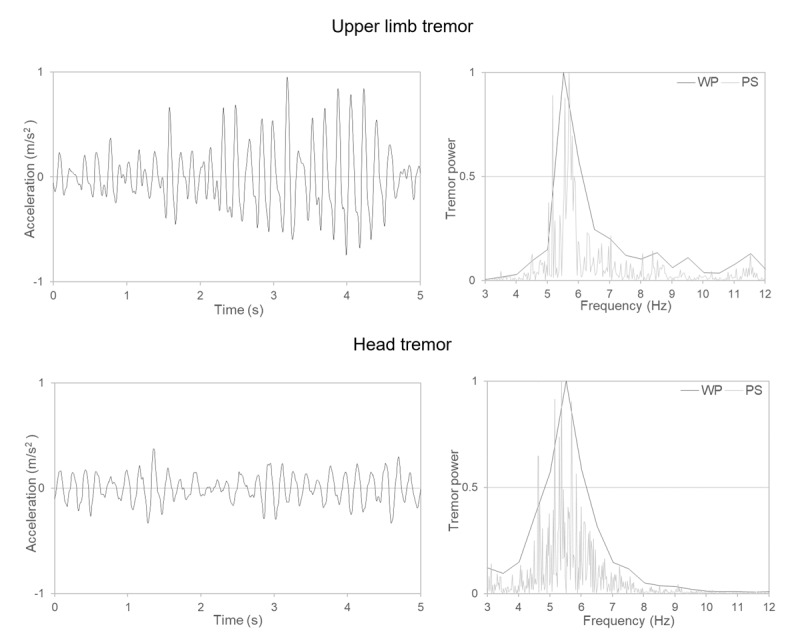
**Kinematic upper limbs and head tremor recordings**. The upper panels depict upper limb postural tremor, while the lower panels represent head tremor. Two five-seconds extracts of accelerometric traces from reference markers in 3D space are shown on the left. On the right, Welch periodograms (WP) overlaid on the power spectra (PS) of the corresponding traces are shown. Note that a clear peak with a small full width half maximum is evident for upper limb postural tremor recording. A narrow peak in the frequency spectrum indicates that much of the oscillation falls within a narrow frequency range, so the movement is characterized by high rhythmicity. This constitutes laboratory support for the identification of the abnormal movement as tremor. The frequency peak was slightly wider for head tremor, given to less regular oscillation and supporting a dystonic component. We did not kinematically record tremor during spiral drawing, where the jerky motor component might have been more evident.

Neurological examination revealed bilateral action tremor of the upper limbs, with a slight left-sided predominance, occasional upper limbs rest tremor, as well as a jerky head tremor, jaw and voice tremor (Video 1). A dystonic posture of the left fifth finger and head dystonia with a tilt to the right were also observed. The patients did not report any sensory trick nor the presence of a null point. Mild bradykinesia, i.e. movement slowness, was noted during repetitive finger movements [6,7,8]. The remainder of neurological examination was unremarkable. Following established protocols, we conducted a tremor kinematic assessment [9,10,11,12]. This confirmed a narrow peak in the frequency spectrum of the upper limb, indicating a high rhythmicity of the abnormal movement, and a wider frequency peak for the head tremor, supporting a dystonic component (Figure 1) [13,14]. Dopamine transporter (DAT) imaging results were normal. Brain magnetic resonance imaging (MRI) revealed periventricular white matter hyperintensities on T2-weighted imaging. A phoniatrist confirmed the presence of vocal tremor with a dystonic component in the vocal A spectrum. A psychiatric evaluation ruled out psychiatric disorders. Propranolol 120 mg, Clonazepam 0.9 mg daily, and Botulinum Toxin A in the neck muscles had beneficial effects on tremor.

**Video 1:** **Neurological examination**. The patient displays a bilateral action tremor in the upper limbs, with a slight left-sided predominance, along with occasional rest tremor. Additionally, she exhibits a jerky tremor affecting the head, accompanied by tremor in the jaw and voice. Furthermore, there is evidence of a dystonic posture in the left fifth finger and a dystonic posture of the head, with a rightward tilt. The patient did not report any sensory trick, nor the presence of a null point. Prolonged eye closure suspicious for blepharospasm can be observed at second 18, 31–33, and 38–39, although the patient did not present a clear hyperactivity of the orbicularis oculi muscles, nor other associated motor manifestations, including apraxia of eyelid opening and increased blink rate. Tremor with a jerky motor component was evident during spiral drawing and when she poured water form one cup to the other. Along with jerky intrusions, mild bradykinesia, i.e., movement slowness, is observed during repetitive finger and hand movements. Despite being slow, her gait appears normal. The remaining neurological examination (not shown in the video) was unremarkable. Please note that the video was recorded under antitremorigenic therapy (Propranolol 120 mg and Clonazepam 0.9 mg daily) and about 4 months after the last injection of Botulinum Toxin type A into the splenius muscles bilaterally.

**Figure 2:**
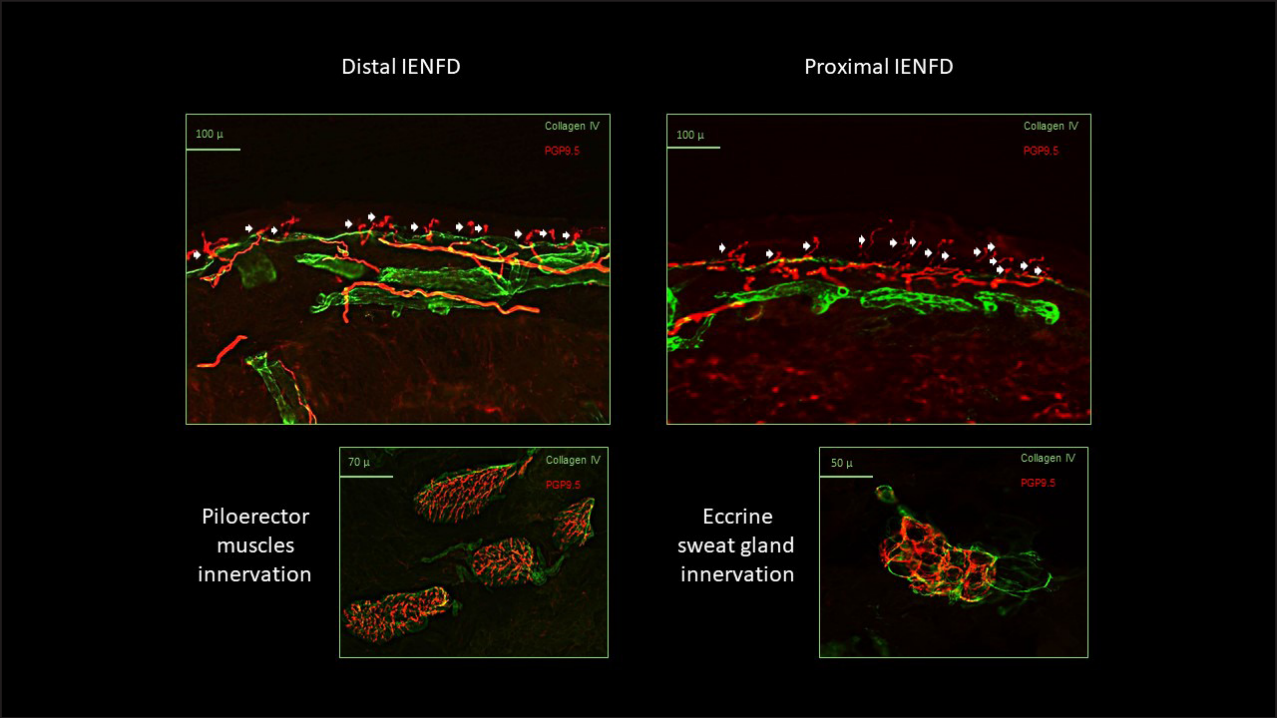
**Skin biopsy**. Exemplificative pictures from the patient’s skin samples, analyzed through indirect immunofluorescence, showing normal intraepidermal nerve fiber density (IENFD) at both distal calf (12 fibers/mm) and proximal thigh (16 fibers/mm). Autonomic innervation was normally represented in dermal annexes, like piloerector muscles and eccrine sweat glands, which could be found only in the distal sample. Red staining represents nerve fibers as marked by antibodies for the pan-axonal marker protein gene product 9.5 (PGP9.5). Green staining represents collagen IV, marking the basal membrane and the connective structure of dermal annexes. White arrows indicate intraepidermal nerve fibers.

In 2012, the patient had undergone surgery and radiotherapy for breast carcinoma and had begun experiencing distal pins-and-needles and burning paresthesia. A nerve conduction study and a lumbar spine MRI returned normal results. Due to suspicion of a small fiber neuropathy, a skin biopsy (Figure 2), quantitative sensory testing, and laser evoked potentials were performed, all resulting normal. An extensive laboratory assessment was performed to rule out the principal polyneuropathy etiologies, including a genetic test for SCN9A, SCN1A, SCN3A, SCN2A, SCN1B, SCN8A, and CACNA1A mutations. The test yielded negative results for the targeted genes. However, it identified an incidental heterozygous variant, c.[1111A>G][=] (p.[Met371Val][=]), in the KCNQ2 gene (NM_004518), potentially related to the patient’s clinical presentation. Genetic testing could not be carried out on the patient’s family members (her father and her father’s sister were deceased at the time of observation). Treatment with Pregabalin was initiated, and clinical improvement was observed. Other relevant information in the patient’s medical history includes two juvenile episodes of transient loss of contact, characterized by a temporary disruption in the patient’s ability to maintain contact and communication without losing consciousness, lasting a few minutes. The episodes occurred suddenly and had a sudden offset, followed by a brief postictal phase characterized by disorientation and confusion. Similar episodes recurred in 2020 when she experienced transient speech disturbances, and in 2023, when she faced difficulty in articulating words. Two subsequent brain MRIs were comparable to the previous one. Electroencephalogram was normal.

## Discussion

We here report a case of a patient with a combined tremor syndrome and a positive family history of tremor who underwent genetic screening for ion channel mutations due to the presence of distally distributed painful symptoms. The screening revealed a heterozygous missense variant, c.[1111A>G] (p.[Met371Val]), in the KCNQ2 gene (chromosome 20q13.33). This gene encodes the voltage-gated potassium-channel subunit KV 7.2 (also known as KCNQ2 protein), which is widely distributed in the nervous system, where it plays a crucial role in controlling neuronal excitability by regulating action potential firing [14,15].

While the KCNQ2 gene is primarily associated with early-onset epileptic encephalopathy, benign familial neonatal seizures and other forms of epilepsy [14,15,16], the variant we identified has not been previously described and is not present in population databases (gnomAD no frequency). However, multiple computational evidence indicate that this missense variant is expected to disrupt KCNQ2 function (Phren Combined Annotation Dependent Depletion -CADD score = 25, Polyphen 0.98) [17,18]. To date, it should be considered a variant of uncertain significance (VoUS) with undefined functional and clinical effects, despite one moderate criterion (PM2) and two supportive (PP2, PP3) for pathogenicity [19]. Although not being diagnosed with epilepsy, the patient reported episodes of loss of contact and difficulty finding words, which are challenging to categorize. Also, with recent evidence suggesting a potential association between KCNQ2 mutations and altered pain perception [20,21], we cannot exclude that the identified VoUS in KCNQ2 may contribute to the distally distributed burning symptoms complained by our patient, even though the identified variant has never been described in patients with neuropathic pain or small fiber neuropathy. Noticeably, our patient did not show any objective evidence of small fiber damage at diagnostic tests, and skin innervation parameters resided within normal values, as shown in Figure 2. However, it is well acknowledged that channelopathies may manifest with small fiber related symptoms due to nerve fibers hyperexcitability, without manifest signs of axonal loss at diagnostic tests [21,22]. Finally, while tremor syndromes have not been recognized as primary symptoms of KCNQ2-related disorders, mutations in other ionic channels have been implicated in autosomal dominant ET and epilepsy susceptibility [2,3,23,24], with individual manifestations varying widely depending on specific mutations, the impact on channel function, and other genetic and environmental factors. In this context, the KCNQ2 could be considered a candidate gene for tremor because it regulates neuronal excitability, synaptic transmission, and depolarization/ hyperpolarization of the membrane [14,15]. Remarkably, most genetic tremor disorders present as combined syndromes [2,4,13], including dystonic syndromes, such as DYT-ANO3, DYT-GCH1, and X-linked dystonia-parkinsonism DYT/PARK-TAF1 (Lubag disease). Furthermore, tremor can occur in specific genetic disorders affecting the nervous system, e.g., spinocerebellar ataxias, fragile X-associated tremor/ataxia syndrome (FXTAS), as well as Wilson’s disease [2,4,13]. However, in some cases, tremor may precede other neurological signs by years or remain the predominant manifestation, potentially mimicking ET, as is the present case. In this regard, while the precise genetic etiology of ET remains elusive, evidence from family and twin studies supports a strong genetic component in its pathogenesis, and several genetic loci have been implicated in familial ET, including FUS, LINGO1, and others [2,3,5,13]. The identification of the KCNQ2 variant raises intriguing possibilities regarding its potential link with the patient’s clinical presentation. Although some factors preclude definitive determination of the mutation’s pathogenicity, i.e., the lack of previously reported associations between KCNQ2 gene mutations and tremor syndromes, as well as the absence of genetic testing in the family members, this case underscores the importance of genetic screening in tremor disorders and pave the way for future genetic case-control studies investigating this association.

## Data Accessibility Statement

The data supporting this study’s findings are available on request from the corresponding author.
